# N-Methyl-D-aspartate (NMDA) and cannabinoid CB_2_ receptors form functional complexes in cells of the central nervous system: insights into the therapeutic potential of neuronal and microglial NMDA receptors

**DOI:** 10.1186/s13195-021-00920-6

**Published:** 2021-11-08

**Authors:** Rafael Rivas-Santisteban, Alejandro Lillo, Jaume Lillo, Joan-Biel Rebassa, Joan S. Contestí, Carlos A. Saura, Rafael Franco, Gemma Navarro

**Affiliations:** 1 Centro de Investigación Biomédica en Red Enfermedades Neurodegenerativas (CiberNed), National Institute of Health Carlos iii, Madrid, Spain; 2 Departament de Bioquímica i Biomedicina Molecular, Universitat de Barcelona, 08028 Barcelona, Spain; 3 Institut de Neurociències, Department de Bioquímica i Biologia Molecular, Universitat Autònoma de Barcelona, Bellaterra, 08193 Barcelona, Spain; 4 Department of Biochemistry and Physiology, Faculty of Pharmacy and Food Science, University of Barcelona, Barcelona, Spain; 5 School of Chemistry, University of Barcelona, Barcelona, Spain

**Keywords:** Alzheimer’s disease, Neuroprotection, G-protein-coupled receptors, Excitotoxicity

## Abstract

**Background:**

The cannabinoid CB_2_ receptor (CB_2_R), which is a target to afford neuroprotection, and N-methyl-D-aspartate (NMDA) ionotropic glutamate receptors, which are key in mediating excitatory neurotransmission, are expressed in both neurons and glia. As NMDA receptors are the target of current medication in Alzheimer’s disease patients and with the aim of finding neuromodulators of their actions that could provide benefits in dementia, we hypothesized that cannabinoids could modulate NMDA function.

**Methods:**

Immunocytochemistry was used to analyze the colocalization between CB_2_ and NMDA receptors; bioluminescence resonance energy transfer was used to detect CB_2_-NMDA receptor complexes. Calcium and cAMP determination, mitogen-activated protein kinase (MAPK) pathway activation, and label-free assays were performed to characterize signaling in homologous and heterologous systems. Proximity ligation assays were used to quantify CB_2_-NMDA heteromer expression in mouse primary cultures and in the brain of APP_Sw/Ind_ transgenic mice, an Alzheimer’s disease model expressing the Indiana and Swedish mutated version of the human amyloid precursor protein (APP).

**Results:**

In a heterologous system, we identified CB_2_-NMDA complexes with a particular heteromer print consisting of impairment by cannabinoids of NMDA receptor function. The print was detected in activated primary microglia treated with lipopolysaccharide and interferon-γ. CB_2_R activation blunted NMDA receptor-mediated signaling in primary hippocampal neurons from APP_Sw/Ind_ mice. Furthermore, imaging studies showed that in brain slices and in primary cells (microglia or neurons) from APP_Sw/Ind_ mice, there was a marked overexpression of macromolecular CB_2_-NMDA receptor complexes thus becoming a tool to modulate excessive glutamate input by cannabinoids.

**Conclusions:**

The results indicate a negative cross-talk in CB_2_-NMDA complexes signaling. The expression of the CB_2_-NMDA receptor heteromers increases in both microglia and neurons from the APP_Sw/Ind_ transgenic mice, compared with levels in samples from age-matched control mice.

**Supplementary Information:**

The online version contains supplementary material available at 10.1186/s13195-021-00920-6.

## Introduction

Alzheimer’s disease (AD) is the most common neurodegenerative disorder affecting more than 46 million people worldwide. The most affected neurons are located in the ascending cholinergic system whose somas are situated in Meinert’s basal nucleus, thereafter, neurodegeneration in hippocampal, amygdala, and neocortex areas leads to the pathological AD features [1–3]. The main excitatory neurotransmitter, glutamate, is crucial for the physiological state of the brain. Excitatory glutamatergic neurotransmission is required for neuronal survival and synaptic plasticity; however, aberrant activity promotes excitotoxicity and cell death [4, 5]. Ionotropic ligand-gated glutamate receptors are the main mediators of glutamate action in the central nervous system (CNS). In addition, glutamate can activate the so-called metabotropic receptors that are not channels but G protein-coupled receptors (GPCRs). Three ionotropic glutamate receptors have been discovered, namely kainate, α-amino-3-hydroxy-5-methyl-4-isoxazolepropionic acid (AMPA), and N-methyl-D-aspartate (NMDA) receptors. The NMDA receptor (NMDAR) plays an important role in neuronal plasticity and learning mechanisms. Memantine, a drug approved for AD therapy [6, 7], targets NMDARs, which are multimers composed of different subunits whose consensual nomenclature is GluN1, GluN2A, GluN2B, GluN2C, GluN2D, GluN3A, and GluN3B [8]. A combination of these subunits leads to different tetrameric functional NMDARs. Moreover, the combination of different subunits leads to NMDARs with different functional and pharmacological properties. Activation of synaptic NMDARs has been reported to control synaptic plasticity and stimulate cell survival, while activation of extrasynaptic NMDARs promotes cell death and thus contributes to the etiology of AD. The limited effect of memantine in AD is likely due to an allosteric effect on extrasynaptic NMDARs [4, 5, 9].

Cannabinoid receptors are widely expressed in the CNS, not only in neurons but also in astrocytes, microglia, and oligodendrocytes. There are two cannabinoid receptors, CB_1_ and CB_2_, that belong to the superfamily of GPCRs. CB_1_ is considered the most abundant GPCR in the CNS and is expressed in many different neuronal types. The expression of the CB_2_ receptor (CB_2_R) is restricted to some neuronal populations, e.g., in the globus pallidus [10] or the cerebellum [11], but is expressed in other neural cell types [12–17]. GPCRs may interact to form homo and heterodimers which for many of the receptors in the superfamily constitute the real functional units [18, 19]. Heteromers have different functionality than homomers, and different heterodimers have different signaling properties thus adding diversity to the action of neurotransmitters/neuromodulators on GPCRs. CB_1_ and CB_2_ receptors may form functional heteromers that are heavily expressed in the neurons of the globus pallidus [10, 20, 21] and that have a relevant function in activated microglia [20, 22]. CB_2_R and CB_1_-CB_2_ receptor heteromers are considered to exert neuroprotective actions [23–27]; they have been proposed as targets to delay progression of Parkinson’s disease [22].

The NMDAR plays a central role in the CNS excitatory neurotransmission, being also a therapeutic target to combat AD. Receptor function deregulation is, at least in part, responsible for the progression of the disease. Based on our previous experience, NMDAR function may be regulated by GPCRs that may, eventually, establish direct interaction with the ionotropic receptor [28–30]. As interest in the CB_2_R is increasing due to its potential to combat neurodegenerative diseases, the aim of this study was to discover CB_2_R-mediated mechanisms of regulation of NMDAR function. We found that NMDAR function can be modulated by interaction with the CB_2_R and that the resulting complexes interact in neurons and microglia in control animals and in AD models.

## Results

### The NMDAR interacts with the CB_2_R in a heterologous expression system

**Fig. 1:**
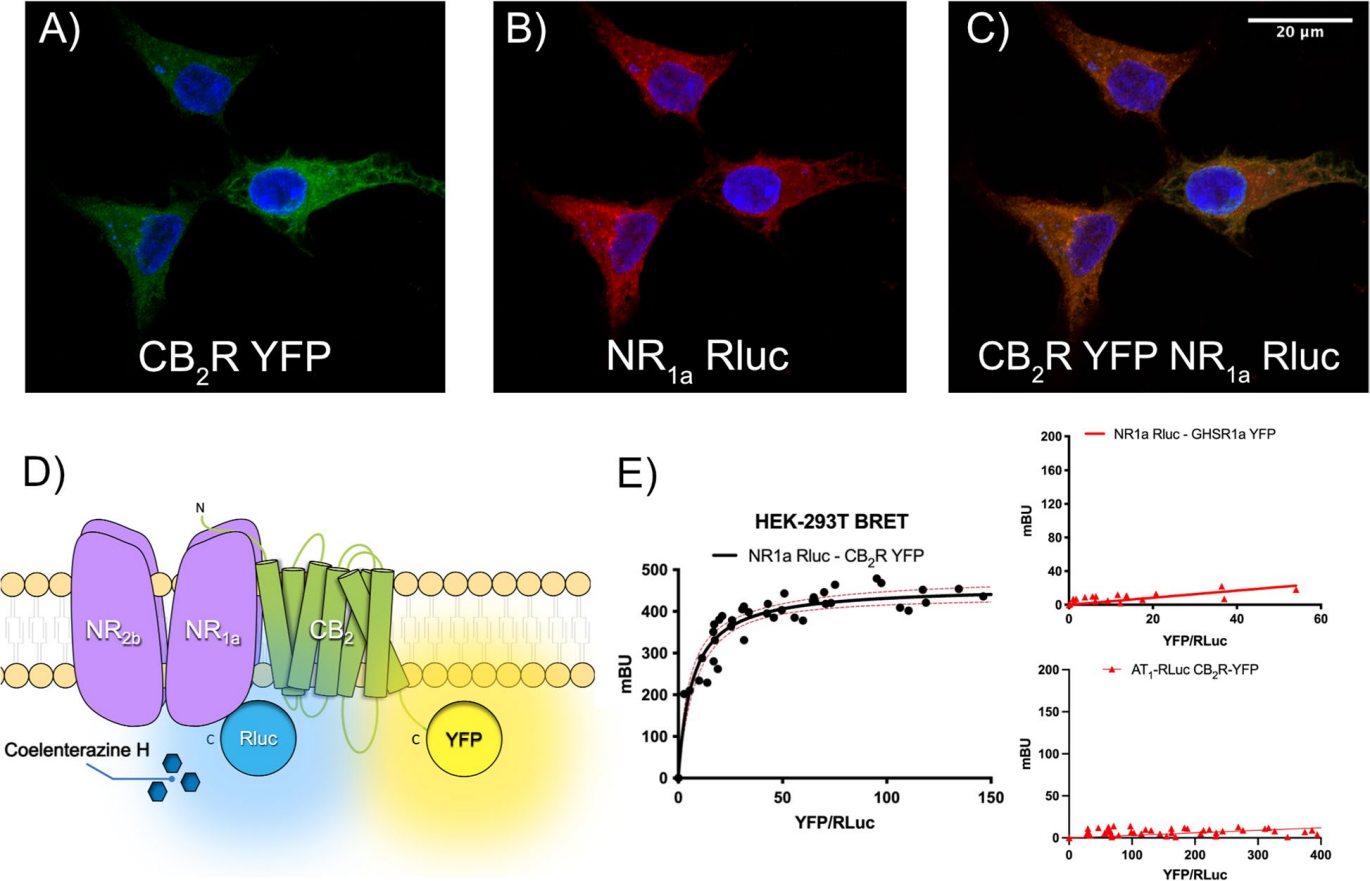
The NMDA receptor interacts with the cannabinoid CB_2_ receptor in a heterologous expression system. **A**–**C** Immunocytochemistry performed in HEK-293T cells expressing CB_2_-YFP (**A**) (1 μg cDNA), that was detected by its own fluorescence (green), and GluN1-Rluc receptor (1 μg cDNA) (**B**), that was detected by a mouse monoclonal anti-Rluc antibody and a secondary Cy3-conjugated anti-mouse IgG antibody (red). Colocalization is shown in yellow (**C**). Cell nuclei were stained with Hoechst (blue). Images are taken near the bottom of the cell, i.e. it mainly includes the membrane in contact with the glass of the plate. Scale bar: 20 μm. **D** Schematic representation of BRET assay: the occurrence of energy transfer depends on the distance between the BRET donor (Rluc) and the BRET acceptor (YFP). **E** BRET assays were performed in HEK-293T cells transfected with a constant amount of cDNAs for GluN1-Rluc (0.25 μg), GluN2B (0.15 μg), and increasing amounts of cDNA for CB_2_R-YFP (0.25 to 1.25 μg) (left) or, as negative controls, using GHSR_1a_R-YFP (0.25 to 1.25 μg) as acceptor  (right top) ), or using  AT_1_R-Rluc (0.5 μg) as donor and CB_2_R-YFP (0.25 to 1.25 μg) as acceptor (right bottom). Values are the mean ± S.E.M. of 6 independent experiments performed in duplicates

The NMDA receptor plays a central role in excitatory neurotransmission, being also a therapeutic target to combat AD. Receptor function deregulation is, at least in part, responsible for the progression of the disease. In this regard, it would be interesting to discover membrane proteins capable of interacting with NMDA receptors and being able to modulate their functionality. We considered that CB_2_Rs could be candidates for receptor-receptor interactions. Accordingly, the human embryonic kidney HEK-293T cell model was transfected with the cDNAs for CB_2_R fused to YFP, for the GluN1 NMDAR subunit fused to Renilla luciferase (RLuc) and for the GluN2B subunit. Expression of GluN1 and GluN2B protomers leads to the assembly of a tetrameric structure, which is required for NMDA receptor functionality. Immunocytochemical assays showed that the fusion protein containing the cannabinoid receptor and the yellow fluorescent protein CB_2_R-YFP, detected by YFP’s own fluorescence, was expressed at the plasma membrane and also intracellularly (Fig. 1A). Qualitatively, similar expression was detected for the NMDA-RLuc receptor by using a specific anti-RLuc antibody (Fig. 1B). Moreover, a high level of colocalization between the two receptors was observed in the plasma membrane and in intracellular organelles (yellow in Fig. 1C). The results are suggestive of possible direct interactions. To demonstrate the hypothesis of a physical interaction between CB_2_ and NMDA receptors, HEK-293T cells were transfected with a constant amount of cDNA for GluN1-RLuc and GluN2B and increasing amounts of cDNA for CB_2_R-YFP. The saturable curve obtained in Bioluminescence Energy Transfer (BRET) experiments was consistent with a physical interacting between CB_2_R and GluN1 and the formation of CB_2_-NMDA receptor complexes (BRET_max_=460 ± 10 mBU BRET_50_ = 10 ± 2; Fig. 1D, E). When HEK-293T cells were transfected with a constant amount of GluN1-RLuc cDNA and increasing amounts of cDNA for the ghrelin GHS1a receptor 1a fused to YFP (GHS-R1a-YFP) or with a constant amount of angiotensin AT_1_ receptor-RLuc cDNA (AT1R-RLuc) and increasing amounts of CB2R-YFP cDNA (CB_2_R-YFP), the linear relationship between the BRET donor/acceptor ratio indicated a lack of interaction of those pair of proteins (negative controls; Fig. 1E right).

### CB_2_R activation impairs signaling via the NMDA receptor expressed in HEK-293T cells

**Fig. 2:**
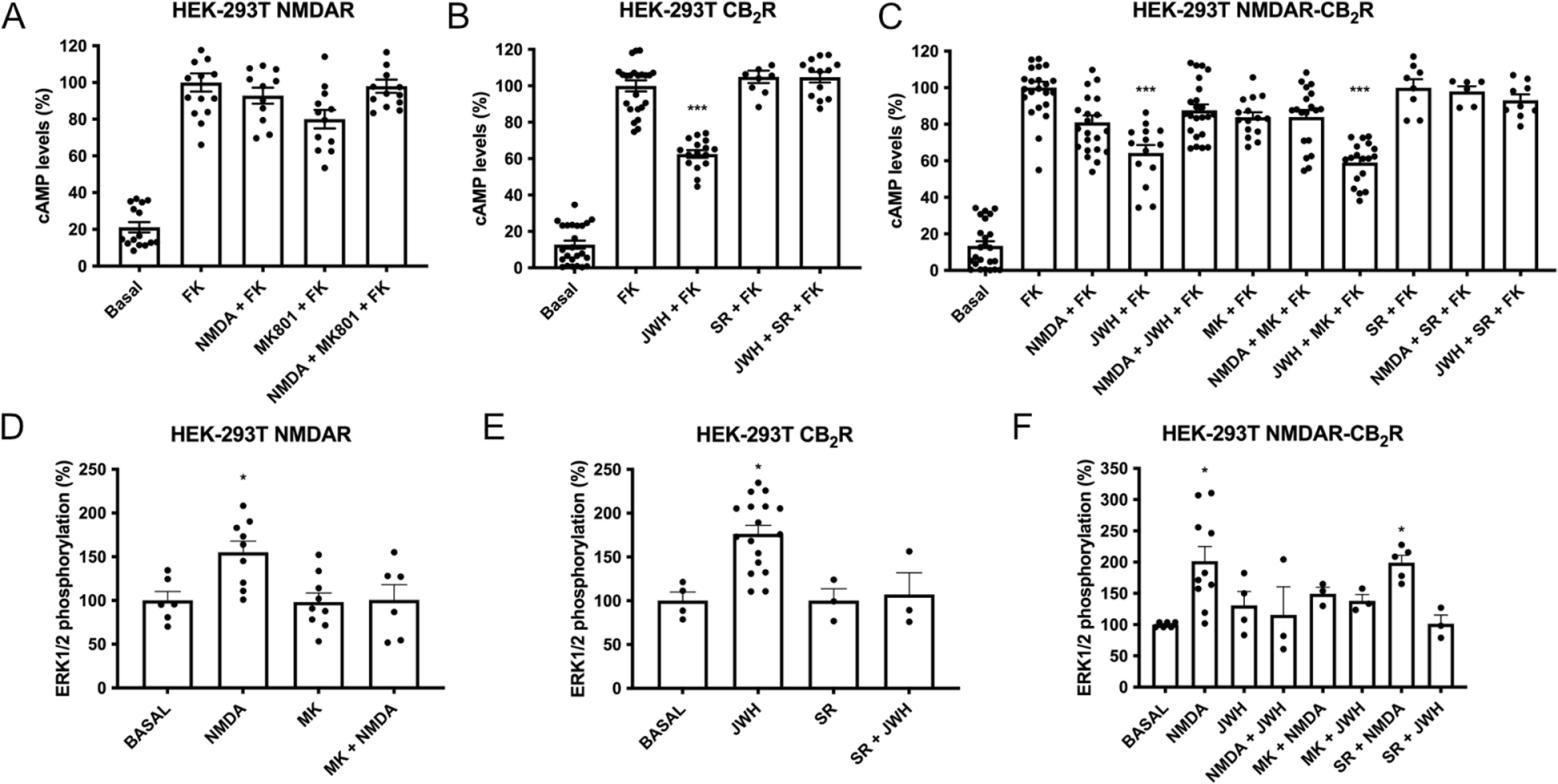
Signaling in HEK-293T cells expressing NMDA-CB_2_R heteromers. HEK-293T cells transfected with the cDNAs for two protomers of the NMDA receptor: GluN1 (1 μg) and GluN2B (0.75 μg) and/or with the cDNA for the CB_2_R (1 μg), and were treated with selective agonists (15 μM NMDA for NMDAR and/or 100 nM JWH-133 for CB_2_R). When indicated cells were pretreated with selective receptor antagonists (1 μM MK-801 for NMDA or 1 μM SR-144528 for CB_2_R). **A**–**C** Intracellular cAMP levels were determined by TR-FRET as described in Methods. As G_i_ coupling was assessed, decreases in cAMP levels were determined in cells previously treated with 0.5 μM forskolin (15 min). Values are the mean ± S.E.M. of 6 independent experiments performed in triplicates. In cAMP one-way ANOVA followed by Bonferroni’s multiple comparison post hoc test were used for statistical analysis (*p < 0.05, **p < 0.01, ***p < 0.001 versus forskolin treatment). ANOVA summary: **A** *F*: 67.6, *p*<0.001; **B** *F*: 238.0, *p*<0.001; **C** *F*: 62.5 *p*<0.001. **D**–**F** ERK1/2 phosphorylation was analyzed using an AlphaScreen®SureFire® kit (Perkin Elmer). Values are the mean ± S.E.M. of 5 independent experiments performed in triplicates. One-way ANOVA followed by Bonferroni’s multiple comparison post hoc test were used for statistical analysis (*p < 0.05, **p < 0.01, ***p < 0.001 versus vehicle treatment). ANOVA summary: **D** *F*: 5.4, *p*<0.005; **E** *F*: 8.3, *p*<0.001; **F** *F*: 3.8, *p*<0.005

In HEK-293T cells only expressing GluN1 and GluN2B subunits, forskolin-stimulated intracellular cyclic-adenylic acid (cAMP) levels were not modified upon treatment with either NMDA or a NMDAR selective antagonist, MK-801 (Fig. 2A). The concentration of NMDA and MK-801was selected according to our previous experience [28–30] and on optimization of dosages based on early reports on NMDAR agonists and antagonists [31, 32]. As G_i_ is the cognate protein coupled to the CB_2_R, activation of the receptor leads to adenylate cyclase activity inhibition and a decrease of intracellular cAMP levels. Such canonical functionality was confirmed in HEK-293T cells expressing CB_2_R treated with forskolin and, afterwards, with a selective CB_2_R agonist, JWH-133. The decrease in forskolin-induced cAMP levels was mediated by the cannabinoid receptor as it was completely counteracted by the pretreatment with a selective antagonist, SR 144528 (Fig. 2B). In HEK-293T cells expressing GluN1, GluN2B and CB_2_R, agonist activation of the cannabinoid receptor produced a significant decrease in forskolin-induced cAMP levels, that was counteracted by the activation of the NMDAR (Fig. 2C). This phenomenon is usually described as negative cross-talk and can serve as a print/pattern to identify CB_2_-NMDA receptor complexes in natural sources. The small decrease upon NMDAR activation was not significant. The CB_2_R antagonist, SR-144528, blocked CB_2_R activation while the NMDAR antagonist produced no effect (Fig. 2C). As a control of specificity, we performed similar assays in cells coexpressing the NMDAR and the ghrelin GHS1a receptor without observing any cross-regulation (Supplementary Figure S1).

Next, we determined mitogen-activated protein kinase (MAPK) pathway activation by means of assays addressing the phosphorylation degree of extracellular signal-regulated kinases, ERK1/2. It should be noted that both NMDAR and CB_2_R activation leads to engagement of the MAPK pathway. Then, in HEK-293T cells expressing GluN1 and GluN2B, NMDA activation induced an effect that was counteracted by MK-801 pretreatment (Fig. 2D); analogously, in HEK-293T expressing the CB_2_R, JWH-133 produced a significant effect that disappeared by the pretreatment with SR-144528 (Fig. 2E). In cells coexpressing the two receptors, NMDA induced a circa 2-fold increase in ERK1/2 phosphorylation; in contrast, CB_2_R activation led to a non-significant response. Furthermore, coactivation with both agonists impeded the link of the CB_2_-NMDA receptor complex to the MAPK pathway (Fig. 2F). Finally, antagonist pre-treatment did not lead to cross-antagonism, i.e., the antagonist of one receptor did not block the effect of the agonist of the partner receptor in the heteromeric complex.

**Fig. 3:**
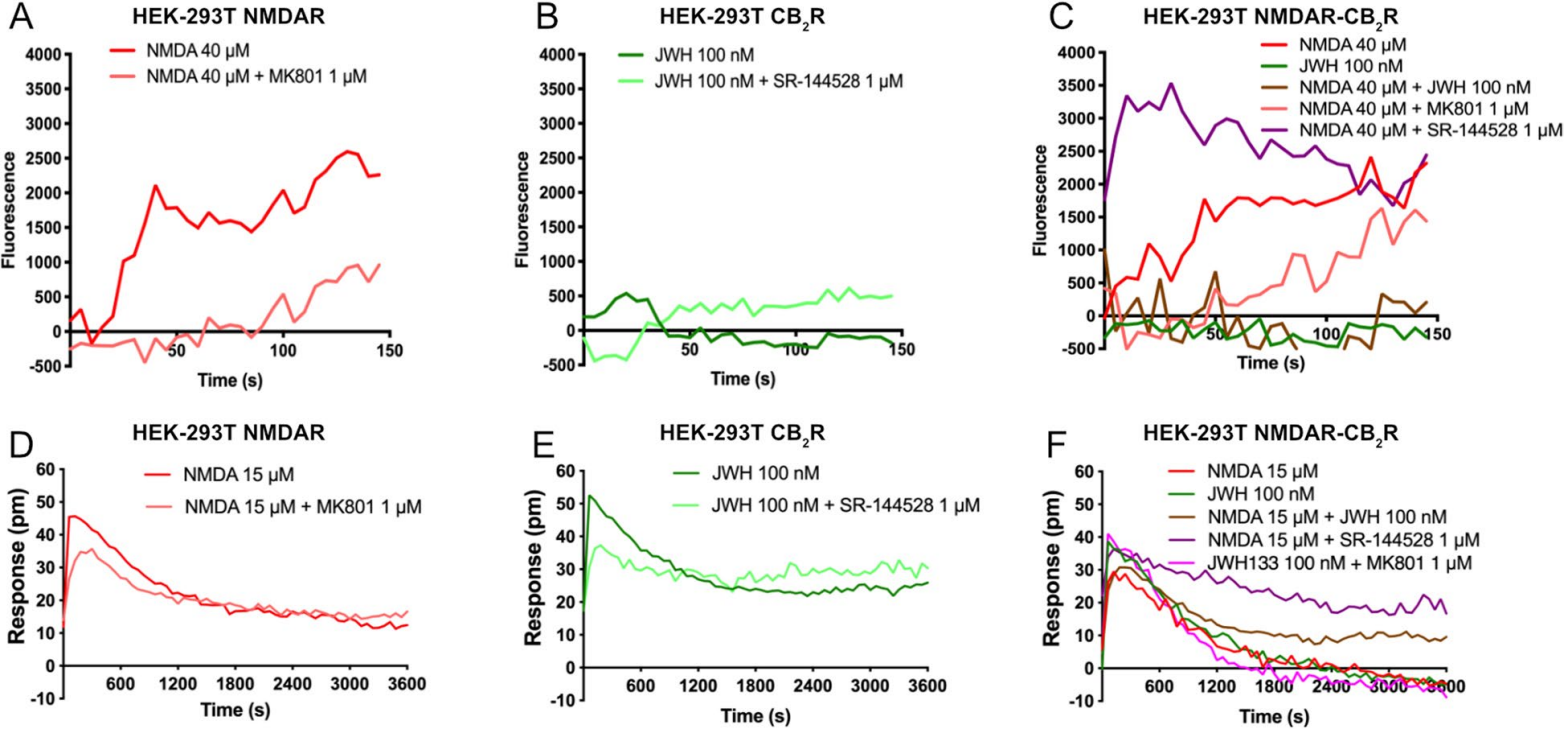
DMR and intracellular calcium mobilization in HEK-293T cells expressing NMDA-CB_2_R heteromers. HEK-293T cells were transfected with the cDNAs for two protomers of the NMDA receptor: GluN1 (1 μg) and GluN2B (0.75 μg) and/or with the cDNA for the CB_2_R (1 μg); for calcium assays, cells were also transfected with the cDNA for the engineered calcium sensor, 6GCaMP (1 μg) (**D**–**F**). Receptors were activated using selective agonists (40 μM NMDA (for calcium detection assays) or 15 μM NDMA (for DMR assays) for NMDAR and/or 100 nM JWH-133 for CB_2_R). When indicated cells were pretreated with selective receptor antagonists (1 μM MK-801 for NMDA or 1 μM SR-144528 for CB_2_R). Real-time calcium-induced fluorescence (**A-C)** or DMR readings (**D-F**) were collected. Values in each figure are from a representative experiment out of 5 independently performed

Activation of NMDA by glutamate results in the opening of the ligand-gated ion channel to allow calcium influx. In HEK-293T cells expressing NMDAR the increase in cytoplasmic calcium caused by NMDA was inhibited in cells pretreated with the antagonist MK-801 (Fig. 3A). As expected, JWH-133 did not produce any calcium signal in HEK-293T cells expressing the CB_2_R (Fig. 3B). However, this CB_2_R agonist blocked the NMDA-induced effect in HEK-293T cells coexpressing NMDAR and CB_2_R, thus showing a negative cross-talk. In addition, the CB_2_R antagonist, SR-144528, potentiated the NMDA-induced effect. These results indicate that, when forming complexes with CB_2_Rs, NMDA receptor activation is restrained by CB_2_R activation and is restored when the  CB_2_R is blocked by its antagonist (Fig. 3C).

Finally, the label-free technique dynamic mass redistribution (DMR) that underscores ligand-induced changes due to multiple pathways and cellular events was applied [33]. First, in cells expressing CB_2_R or NMDAR redistribution of mass occurred upon agonist treatment (Fig. 3D, E), the effects were partially inhibited by pretreatment with selective antagonists. In cotransfected cells, it was demonstrated that coactivation induced no additive effect, while the CB_2_R antagonist, SR-144528, potentiated NMDA activation (Fig. 3F).

To sum up, heteromers constituted by the two receptors (CB_2_-NMDA-Hets) show a negative cross-talk that may disappear when the partner receptor is blocked by an antagonist. It should be noted that the blockade of CB_2_R markedly potentiates the ligand-gated ionotropic action subsequent to NMDAR activation.

### CB_2_R activation blocks NMDA signaling in activated microglia

**Fig. 4:**
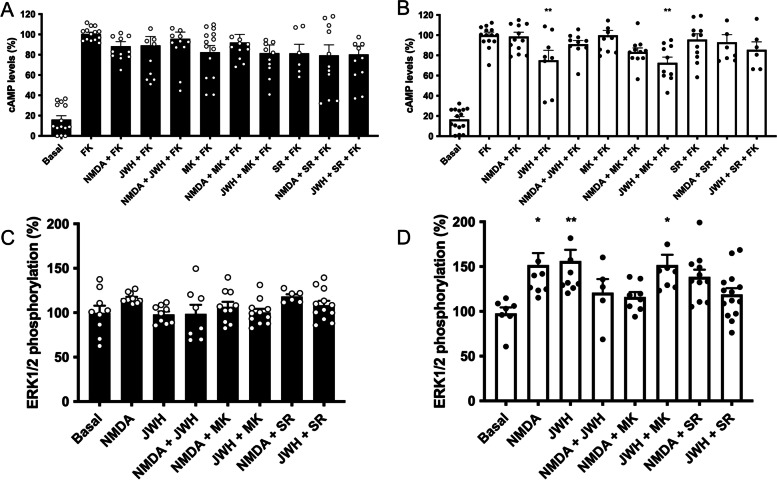
Signaling in primary microglia activated with LPS and IFN-γ. Primary microglial cells were incubated for 48 h in the absence (black bars) or in the presence (white bars) of 1 μM LPS and 200 U/mL IFN-γ. Cells were treated with selective agonists (15 μM NMDA for NMDA channel, and/or 100 nM JWH-133 for CB_2_R) and cAMP levels and MAPK pathway activation were determined. As G_i_ coupling was assessed, decreases in cAMP levels were determined in cells previously treated with 0.5 μM forskolin (15 min). When indicated cells were pretreated with selective receptor antagonists (1 μM MK-801 for NMDA or 1 μM SR-144528 for CB_2_R). Values are the mean ± S.E.M. of 5 independent experiments performed in triplicates. One-way ANOVA followed by Bonferroni’s multiple comparison post hoc test were used for statistical analysis (*p < 0.05, **p < 0.01, ***p < 0.001 versus forskolin treatment in cAMP determinations or versus vehicle treatment (basal) in ERK phosphorylation assays). ANOVA summary: **A** *F*: 12.0, *p*<0.001; **B** *F*: 30.0, *p*<0.001, **C** *F*: 1.8, *p*<0.093, **D** *F*: 4.1, *p*<0.001

The cannabinoid receptor CB_2_R is upregulated in activated microglia, thus being a promising target for neuroprotection. Then, to evaluate the possible role of the CB_2_R in regulating NMDAR function, mouse primary microglia were activated (48 h) with 1 μM LPS and 200 U/ml IFN-γ and treated with receptor ligands. Treatment of non-activated cells with the CB_2_R agonist, JWH-133, did not produce any significant decrease of forskolin-induced cAMP levels (Fig. 4A). This is likely due to the low CB_2_R expression levels in resting microglia. In contrast, in activated microglial cell cultures, in which the CB_2_R is upregulated, JWH-133 treatment induced a significant decrease of forskolin-induced cAMP levels which was completely blocked by the CB_2_R antagonist, SR144528, but not by NMDAR antagonist, MK-801. However, no effect was found when the two agonists were added together, i.e., a negative cross-talk was detected (Fig. 4B). These results were similar to those observed in transfected HEK-293T cells.

In resting cells the effect on MAPK pathway activation of either JWH-133 or NMDA was not significant (Fig. 4C). In activated microglia, the significant effect of both CB_2_R and NMDAR agonists on increasing ERK1/2 phosphorylation was, however, significantly decreased when the two agonists were added together (Fig. 4D). Pretreatment with the NMDAR antagonist, MK-801, did not block the effect of JWH-133, whereas the CB_2_R antagonist, SR-144528, slightly decreased the NMDAR-mediated effect. These results are consistent with the occurrence of CB_2_-NMDA-Hets in activated microglia in which CB_2_R activation exerts a negative regulation over the NMDAR link to the MAPK pathway.

### Differential levels of CB_2_-NMDA-Hets in neurons and microglia from APP_Sw/Ind_ mice

**Fig. 5:**
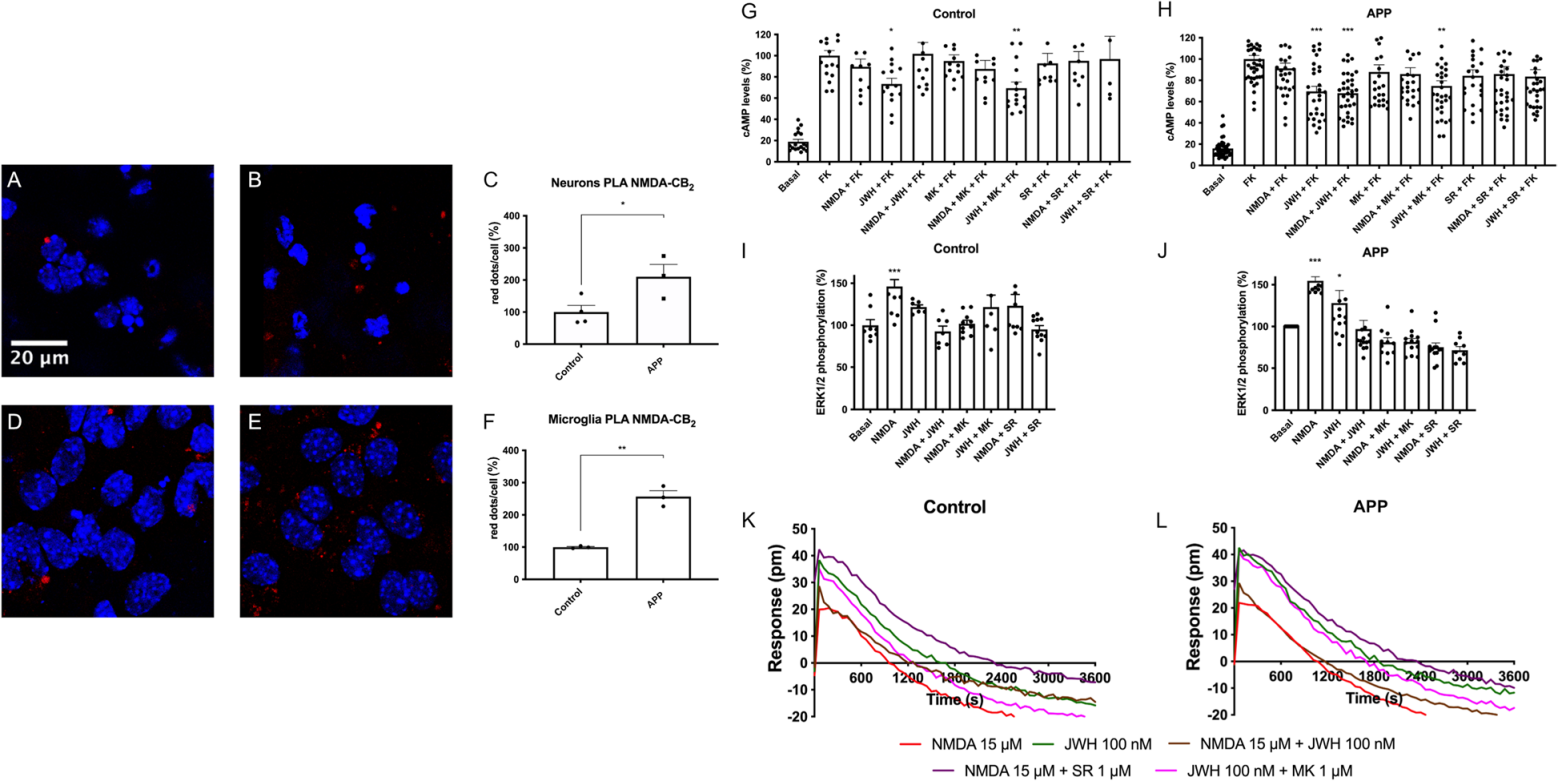
NMDA-CB_2_R heteromer levels and functionality in APP_Sw/Ind_ neurons. **A**–**F** Expression of NMDA-CB_2_R heteromers in mouse primary neurons (**A**, **B**) and microglia (**D**, **E**) of wild type (**A**, **D**) and APP_Sw/Ind_ transgenic mice (B, E) as determined by PLA (see Materials and Methods) using specific primary antibodies against the GluN1 subunit and against the CB_2_R receptor. Confocal microscopy images (stacks of 3 consecutive panels) show heteroreceptor complexes as red clusters over Hoechst-stained nuclei (blue). Three independent experiments were performed using, for each condition, 5 preparations per session. Bar graphs show the amount of red dots/cell in APP_Sw/Ind_ mice and control animals (*p < 0.05, **p < 0.01; Student’s *t* test versus the control condition). **G**–**L** Primary neurons from control and APP_Sw/Ind_ mice were treated with selective agonists (15 μM NMDA for NMDA channel, and/or 100 nM JWH-133 for CB_2_R) and cAMP levels (**G**, **H**), ERK1/2 phosphorylation (**I**, **J**), and DMR (**K**, **L**) assays were determined. When indicated cells were pretreated with selective receptor antagonists (1 μM MK-801 for NMDA or 1 μM SR-144528 for CB_2_R). Values are the mean ± S.E.M. of 5 independent experiments performed in triplicates. One-way ANOVA followed by Bonferroni’s multiple comparison post hoc test were used for statistical analysis (*p < 0.05, **p < 0.01, ***p < 0.001 versus forskolin treatment in cAMP determinations or versus vehicle treatment (basal) in ERK phosphorylation assays). ANOVA summary: **G** *F*: 14.5, *p*<0.001; **H** *F*: 25.3, *p*<0.001, **I** *F*: 5.6, *p*<0.001, **J** 13.2, *p*<0.001

Finally, we investigated the levels and cross-talk of CB_2_R/NMDAR complexes in primary hippocampal neurons of control and APP_Sw/Ind_ mice, a transgenic line that expresses the human amyloid precursor protein (APP) harboring the Indiana and Swedish mutations. First, the expression of CB_2_-NMDAR-Hets was determined by in situ PLA (see the “Materials and Methods” section). Compared with control mice, CB_2_-NMDA-Hets expression was circa two-fold higher in primary neurons and circa 2.6-fold higher in microglia from APP_Sw/Ind_ mice (Fig. 5A–F). These results demonstrate that CB_2_R-NMDA receptor complexes are aberrantly increased in primary neurons and microglia of APP_Sw/Ind_ mice.

### CB_2_R activation blocks NMDAR-mediated signaling in primary hippocampal neurons from APP_Sw/Ind_ mice

Intracellular cAMP levels were determined in primary neurons treated with forskolin and with selective receptor agonists. Whereas CB_2_R activation produced a significant 30% decrease of forskolin-induced cAMP levels, NMDA did not generate any significant effect but counteracted the activation of the cannabinoid receptor (Fig. 5G). Thus, the negative cross-talk observed in transfected HEK-293T cells was also noticeable in primary neurons, thus suggesting the occurrence of functional CB_2_-NMDA-Hets in hippocampal neurons. A similar phenomenon was also observed upon analysis of MAPK pathway activation: JWH-133 and NMDA induced ERK1/2 phosphorylation, which was undetectable in cells simultaneously treated with the two agonists (Fig. 5I). In addition, as observed in HEK-293T cells, pretreatment with the CB_2_R selective antagonist blocked the JWH-133-induced signal while exerting no significant effect on NMDAR activation. Reciprocally, the NMDAR antagonist reverted the effect of NMDA but not that due to JWH-133. Finally, similar results were obtained in DMR label-free assays (Fig. 5K), i.e., NMDA receptor activation blocked CB_2_R function and vice versa. Moreover, as observed in transfected HEK-293T cells, SR144528 pretreatment potentiated the NMDAR action.

Finally, we investigated the expression and cross-talk of cannabinoid CB_2_ and NMDA receptors in primary neurons from control and APP_Sw/Ind_ mice. Interestingly, it was observed that CB_2_R activity was potentiated in APP_Sw/Ind_ neurons, and contrary to control mice, it was not blocked by cotreatment with NMDA (see Fig. 5H). Upon analysis of MAPK pathway activation, JWH-133 and NMDA induced ERK1/2 phosphorylation, which was undetectable in cells simultaneously treated with the two agonists (Fig. 5J). Remarkably, the pretreatment with the CB_2_R selective antagonist blocked the NMDAR-mediated effect in samples from control animals, and with more potency than in APP_Sw/Ind_ neurons. This cross-antagonism was found in the opposite direction, i.e. the antagonist of the NMDAR completely blocked the JWH-133 effect. Finally, in dynamic mass redistribution assays, a negative cross-talk was identifiable when the two agonists were added together. This phenomenon, which was observed in neurons from both control and APP_Sw/Ind_ mice (Fig. 5L), indicates that NMDAR activation blocks CB_2_R function and vice versa.

### CB_2_-NMDA-Hets expression increase in cortical slices from APP_Sw/Ind_ compared to those from age-matched mice

**Fig. 6:**
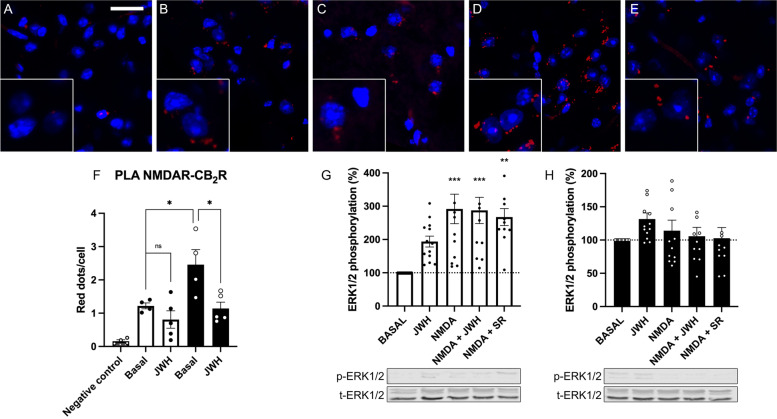
NMDA-CB_2_R heteromer levels and functionality in adult APP_Sw/Ind_ mice brain slices. **A**–**F** Expression of NMDA-CB_2_R heteromers in adult brain (brain cortex) slices from control (**B**, **C**) and APP_Sw/Ind_ transgenic mice (**D**, **E**) as determined by PLA (see the “Materials and Methods” section) using specific primary antibodies against the GluN1 subunit and against the CB_2_R receptor. For negative control, only the anti-GluN1 antibody was used (**A**). Confocal microscopy images (stacks of 3 consecutive panels) show heteroreceptor complexes as red clusters; nuclei were stained with Hoechst (blue). Three independent experiments were performed using, for each condition, 5 preparations per session. Scale bar: 40 μm. Bar graphs (**F**) show the amount of red dots/cell in APP_Sw/Ind_ mice and control animals (*p < 0.05; One-way ANOVA followed by Bonferroni’s multiple comparison *p*ost hoc test were used for statistical analysis). MAPK phosphorylation assays were performed in control (**G**) and APP_Sw/Ind_ (**H**) transgenic mice brain slices. Slices were treated with selective agonists (15 μM NMDA, and/or 100 nM JWH-133). When indicated, slices were pretreated with the CB_2_R selective receptor antagonist (1 μM SR-144528). Results are expressed as a percentage over basal and are the extracellular signal regulated (ERK) 1/2 phosphorylation mean ± SEM signals of three independent experiments performed in triplicates. ANOVA summary: **F** *F*: 10.4, *p*<0.001, **G** *F*: 7.7, *p*<0.001, **H** *F*: 1.2 , n.s

As neuronal cultures may change the phenotype upon a time in culture, we wanted to assess in cortical slices of adult mice the expression and functionality of the CB_2_-NMDA-Hets, with special focus in the APP_Sw/Ind_ AD model. The PLA results (Fig. 6A–F) demonstrated a higher expression of the CB_2_-NMDA-Het in slices from adult APP_Sw/Ind_ mice (compared with those from age-matched control mice). On the other hand, treatment with the selective CB_2_R agonist, JWH-133, lead to a marked decrease in CB_2_-NMDA-Het expression, not only in APP_Sw/Ind_ slices but also in those from control mice. Functionality was assessed by means of assays of ERK1/2 phosphorylation in adult mice to find, in slices from control animals, that both JWH-133 and NMDA led to MAPK pathway activation and that there was neither an additive effect nor cross-antagonism (Fig. 6G). In contrast, the MAPK pathway was slightly activated when slices from APP_Sw/Ind_ mice brain were incubated with JWH-133. The slices were refractory to the action of exogenous NMDA, irrespective of the presence or absence of JWH-133 (Fig. 6H). These results indicate that the link of the NMDAR to the MAPK pathway is blocked in brain cortical slices of adult APP_Sw/Ind_ mice.

## Discussion

Ionotropic glutamate receptors are essential for the proper functioning of the mammalian CNS. The NMDA receptor is key to many aspects of glutamate-mediated actions in the nervous system, for excitatory neurotransmission, but also for development and neurogenesis. However, a dark side is related to excitotoxicity due to excessive levels of extracellular glutamate and the subsequent accumulation of toxic levels of ions in the cytoplasm of neurons. In this sense, it was hypothesized that NMDA would be a target of neurodegenerative diseases since, in fact, among the few existing drugs to combat AD, a negative allosteric NMDAR modulator, Memantine, was approved several years ago. Although the main interest has logically focused on neurons, NMDARs are expressed in glial cells, where they play a critical role in maintaining brain homeostasis. This work was based on the hypothesis that the action of NMDAR could be regulated by cannabinoid receptors and we focused on neurons and microglia, whose activation phenotype affects the progression of AD.

Although the different GPCRs tend to form dimers and oligomers, it was assumed that the multimeric structure of ionotropic receptors prevented the addition of more proteins to form macromolecular complexes with particular physiological properties. In fact, few examples of direct interactions between ionotropic receptors and GPCRs have been reported. We had previously demonstrated the interaction of a GPCR, which is a target for neuroprotection, the adenosine A_2A_ receptor, and NMDAR. This complex appears to have a relevant role in activated microglia where these complexes, which are expressed in the microglia of WT animals, are markedly upregulated in cells of AD transgenic mice [29].

In this report, we first addressed the possibility of a direct interaction between CB_2_R and NMDAR, and the results were positive, that is, these two receptors can form complexes that alter the effect exerted by NMDA or CB_2_R agonists. This finding is remarkable and confirms that GPCRs that are relevant to maintaining a correct neuroprotective balance (the adenosine A_2A_ receptor is a significant example ([29]) can interact with ionotropic receptors. Although CB_2_R is less abundant in neurons than the cannabinoid CB_1_ receptor, it can be found in neurons in different brain regions, and here we were able to find CB_2_-NMDA-Hets in primary hippocampal neurons (Fig. 5). In microglia, heteromers were present but at a lower level at resting than in activated cells; this is likely due to the increase in CB_2_R, whose expression in resting conditions was quite low.

In the heterologous expression system, the most noticeable property of the CB_2_-NMDA-Hets was the negative cross-talk, namely simultaneous treatment with the two agonists led to the absence of response in either the Gi/adenylate cyclase/cAMP pathway or the MAPK pathway. Because CB_2_R agonists could not, in the heteromeric context, significantly phosphorylate ERK1/2, the blocking effect appears to be direct, that is, due to intra-CB_2_-NMDA-Het allosteric interactions and conformational changes upon binding of cannabinoids to the CB_2_R. Perhaps the most relevant effect was the reduction by CB_2_R agonists of the ionotropic function of the NMDAR. The cross-antagonism found in complexes formed by two GPCRs was not found in HEK-293T cells expressing CB_2_ and NMDA receptors. This contrast with the A_2A_R-NMDAR properties whose structure allows detecting cross-antagonism when GPCR-GPCR heteromer formation is suspected [19, 34, 35].

Importantly, CB_2_-NMDA-Hets were also detected in hippocampal neurons, although the glutamate/cannabinoid relationships are more complex. In WT animals, the findings related to Gi-coupled actions of the CB_2_R were similar to those found in the heterologous cells; basically, there was a negative cross-talk. However, this cross-talk was not found in cells from the APP_Sw/Ind_ transgenic mice. On the one hand, these findings show that hippocampal neurons from WT and transgenic mice are different, already, in the early steps of CNS development; the AD-like phenotype takes months to be detectable. On the other hand, the results may indicate a lack of complexes in neurons from the AD mouse model. The cross-antagonism detected in samples from those mice, i.e., the antagonist of one receptor blunted the link to the MAPK pathway and vice versa, shows that CB_2_-NMDA-Hets are present. Thus the most reasonable hypothesis is that different populations of receptors coexist and that the CB_2_-NMDA-Het is one of them. This hypothesis could explain why NMDA does not affect the JWH-133 effect on cAMP levels; perhaps Gi-coupled to CB_2_R are not interacting with NMDARs in neurons from transgenic animals or the CB_2_-NMDA-Het is not well coupled to the Gi. Remarkably, the negative cross-talk in the link to the MAPK pathway occurs in both neurons from WT and from APP_Sw/Ind_ animals. The presence of complexes was confirmed by PLA, which furthermore showed that the expression of the CB_2_-NMDA-Het increases in both microglia and neurons from the transgenic mice (compared with levels in WT mice).

NMDAR is a target to combat AD. However, drugs that directly affect its function are not effective in the medium/long term [7]. Finding GPCRs that can interact and modulate NMDAR-mediated function holds promise for innovative treatments targeting neurons, microglia, or both. In the case of A_2A_R, impairment of NMDAR function by A_2A_R antagonists is an attractive possibility. In the present study, cannabinoids could provide equivalent benefits by significantly reducing the effect of agonists that activate NMDAR. With the exception of Δ^9^-tetrahydrocannabinol (THC), which produces CB_1_R-mediated psychotropic effects, most of the natural cannabinoids studied so far are generally safe. Additionally, there is an increased interest in cannabinoids as potential drugs to combat a variety of diseases [36–38].

A deleterious factor in neurodegenerative diseases, including AD, is excitotoxicity, that is, the aberrant increase in cytoplasmic Ca^2+^ levels after excessive stimulation of NMDAR by extracellular glutamate [39]. Since allosteric modulators that act directly on NMDAR do not provide much help to AD patients, an interacting GPCR-mediated allosteric modulation is an attractive possibility to explore further. In the case of CB_2_R, its complex pharmacology can be an added value to find the best way to regulate the NMDAR function. In fact, cannabinoids show multiple modes of binding and biased signaling due to the wormhole-like structure of their orthosteric site and due to the existence of various non-orthosteric binding sites. Multiple modes of cannabinoid binding to CB_2_R lead to specific receptor conformations underlying functional selectivity (biased agonism) [40] and, ultimately, differentially regulating NMDAR function. As an example, we have designed bitopic ligands that bind to an exosite located at the entrance of the structure that connects the orthosteric site with the lipid bilayer [41]. These findings constitute a selective advantage since the expression of CB_2_-NMDA-Het increases in neurons and microglia of APP_Sw/Ind_ transgenic mice.

### Study limitations

The main and most common limitation is related to the AD transgenic model. There is not any model that could appropriately cover the non-familial cases of the disease. Another limitation is that modulation cannot be tested in human neurons and that the occurrence of the dimer has to be confirmed using human brain samples. However, the APP_Sw/Ind_ transgenic mice is one of the most used animal models of AD. The results of the study strongly indicate that cannabinoids negatively modulate the NMDAR function like memantine does. Then it would be possible to design a clinical trial looking for extra efficacy of one of the known safe cannabinoids acting on CB_2_ receptors when combined with memantine.

## Conclusions

In conclusion, the present study demonstrates that CB_2_ and NMDA receptors form CB_2_R-NMDAR complexes that are expressed in cells of the CNS. CB_2_R-NMDAR complexes are novel functional units with singular signaling properties. Particularly, activation of the cannabinoid receptor reduces the signaling output of NMDA. In addition, the level of expression of the CB_2_-NMDA complexes increases in both microglia and neurons from the APP_Sw/Ind_ transgenic mice AD model, compared with levels in WT mice.

## Materials and methods

### Reagents

Lipopolysaccharide (LPS), interferon-γ (IFN-γ), JWH-133 (JWH), and SR-144528 (SR) were purchased from Sigma-Aldrich (St Louis, MO, USA). N-Methyl-D-aspartate (NMDA), MK-801 (MK), and forskolin (FK) were purchased from Tocris (Bristol, UK). Tau and p-Tau proteins were kindly provided by Prof. J. Avila (CBM, UAM-CSIC, Madrid, Spain). Detailed descriptions of the elaboration and processing of proteins can be found elsewhere [42, 43]

### HEK-293T cells and primary cultures

Human embryonic kidney HEK-293T (lot 612968) cells were acquired from the American Type Culture Collection (ATCC). They were amplified and frozen in liquid nitrogen in several aliquots. Cells from each aliquot were used until passage 12.

HEK-293T cells were grown in Dulbecco’s modified Eagle’s medium (DMEM) (Gibco) supplemented with 2 mM L-glutamine, 100 μg/ml sodium pyruvate, 100 U/ml penicillin/streptomycin, MEM non-essential amino acids solution (1/100), and 5% (v/v) heat-inactivated fetal bovine serum (FBS) (all supplements were from Invitrogen, Paisley, Scotland, UK). Cells were maintained at 37 °C in a humid atmosphere of 5% CO_2_.

To prepare mice hippocampal primary microglial cells, the brain was removed from C57BL/6 mice of 2–4 days of age. Microglial cells were isolated as described in [44]. Briefly, the brain was dissected, carefully stripped of its meninges and the hippocampus was digested with 0.25% trypsin for 20 min at 37 °C. Trypsinization was stopped by washing the tissue. Cells were brought to a cell suspension by passage through 0.9 mm and 0.5 mm nails followed by passage through a 100 μm pore mesh. Glial cells were resuspended in medium and seeded at a density of 1 × 10^6^ cells/ml in 6-well plates for cyclic adenylic acid (cAMP) assays, in 12-well plates with coverslips for in situ proximity ligation assays (PLA) and in 96-well plates for mitogen-activated protein kinase (MAPK) activation experiments. Cultures were grown in DMEM medium supplemented with 2 mM L-glutamine, 100 U/ml penicillin/streptomycin, MEM non-wssential amino acids preparation (1/100) and 5% (v/v) heat-inactivated fetal bovine serum (FBS) (Invitrogen, Paisley, Scotland, UK) and maintained at 37°C in humidified 5% CO_2_ atmosphere and, unless otherwise stated, medium was replaced once a week.

For culturing primary neurons, the hippocampus from mouse embryos (E19) was removed and the neurons were isolated as described by [45] [45] and plated at a density of circa 120,000 cells/cm^2^. Cells were grown in a neurobasal medium supplemented with 2 mM L-glutamine, 100 U/mL penicillin/streptomycin, and 2% (v/v) B27 supplement (Gibco) in a 6-, 12- or 96-well plate for 12 days. Cultures were maintained at 37^o^C in a humidified 5% CO_2_ atmosphere and the medium was replaced every 4–5 days.

Immunodetection of specific markers (Neu N for neurons and CD-11b for microglia) showed that neuronal preparations contained >98% neurons and microglia preparations contained, at least, 98% microglial cells [28].

### Preparation of brain cortex slices

 Mouse brains were extracted in a cold chamber at a temperature of 4°C. Brain slices (BS) with a thickness of 500 μm were made with the aid of a mouse coronal matrix (Agnthos, ref. 69-2165) and subsequently, the cortical region was isolated. Brain slices were maintained in Krebs’s buffer (124 mM NaCl, 4 mM KCl, 1,25 mM KH_2_PO_4_, 1,5 mM MgSO_4_, 10 mM glucose, 26 mM NaHCO_3_, 1,5 mM CaCl_2_ and carbogen). Brain slices were incubated for 2 h at 32 °C in a humidified 5% CO_2_ atmosphere, Krebs’s buffer was replaced once in the first 30 min. After that, BSC were treated or not for 15 min with the selective antagonist for CB_2_R (SR-144528 (1 μM)) followed by 15 min treatment with the selective agonists (NMDA (15 μM) and/or JWH-133 (100 nM)). After treatment, the slices were immediately frozen in dry ice to stop the metabolic activity.

On the one hand, ERK1/2 phosphorylation was determined by Western blot. Samples were sonicated on ice with 10-s pulse, 20-s rest, and 10-s pulse, using lysis buffer for tissue disaggregation and cell lysis. Final lysates total protein was adjusted to 2 μg/μL with SDS and lysis buffer using BCA quantification. Equivalent amounts of protein (40 μg) were subjected to electrophoresis (10% SDS-polyacrylamide gel) and transferred onto PVDF membranes (Immobilon-FL PVDF membrane, MERK, St. Louis, MO, USA) for 30 min using Trans-Blot Turbo system (Bio-Rad). Then, the membranes were blocked for 2 h at room temperature (constant shaking) with Odyssey Blocking Buffer (LI-COR Biosciences, Lincoln, NE, USA) and labeled with a mixture of primary mouse anti-phospho-ERK 1/2 antibody (1:2500, MERK, Ref. M8159), primary rabbit anti-ERK 1/2 antibody (1:40,000, MERK, Ref. M5670), which recognizes both phosphorylated and non-phosphorylated ERK1/2 overnight at 4 °C with shaking. Then, the membranes were washed three times with PBS containing 0.05% tween for 10 min and subsequently were incubated by the addition of a mixture of IRDye 800 anti-mouse antibody (1:10,000, MERK, Ref. 92632210) and IRDye 680 anti-rabbit antibody (1:10,000, MERK, Ref. 926-68071) for 2 h at room temperature, light-protected. Membranes were washed 3 times with PBS-tween 0.05% for 10 minutes and once with PBS and left to dry. Bands were analyzed using Odyssey infrared scanner (LI-COR Biosciences). Band densities were quantified using Fiji software, and the level of phosphorylated ERK1/2 was normalized using the total ERK 1/2 protein band intensities. Results obtained are represented as the percent over basal (non-stimulated cells).

### APP_Sw/Ind_ transgenic mice

APP_Sw/Ind_ transgenic mice (line J9; C57BL/6 background) expressing the human APP695 harboring the FAD-linked Swedish (K670N/M671L) and Indiana (V717F) mutations under the platelet-derived growth factor subunit B (PDGFβ) promoter were obtained by crossing heterozygous APP_S__w/Ind_ to non-transgenic (control) mice [46]. Control and APP_S__w;Ind_ embryos (E16.5) and adult mice (6 months) were genotyped individually, and hippocampus/cortex dissected and prepared for microglia and neuron primary cultures as described elsewhere [22, 28]. All experimental procedures were conducted according to the approved protocols from the Animal and Human Ethical Committee of the Universitat Autònoma de Barcelona (CEEAH 2895) and Generalitat de Catalunya (10571) following the experimental European Union guidelines and regulations (2010/63/EU)

### Fusion proteins

Human cDNAs for the GluN1 NMDA receptor subunit, for the CB_2_ receptor, and for the ghrelin GHS1a receptor, all cloned into pcDNA3.1 were amplified without their stop codons using sense and antisense primers harboring either BamHI and HindIII restriction sites to amplify GluN1, BamHI, and KpnI restriction sites to amplify CB_2_ receptor or EcoRI and KpnI restriction sites to amplify GHS1a receptor. Amplified fragments were then subcloned to be in frame with an enhanced yellow fluorescent protein (pEYFP-N1; Clontech, Heidelberg, Germany) or a RLuc (pRLuc-N1; PerkinElmer, Wellesley, MA) on the C-terminal end of the receptor to produce GluN1-RLuc, CB_2_R-YFP, and GHSR1a-YFP fusion proteins.

### Cell transfection

HEK-293T cells were transiently transfected with the corresponding cDNA by the PEI (PolyEthylenImine, Sigma-Aldrich) method. Briefly, the corresponding cDNA diluted in 150 mM NaCl was mixed with PEI (5.5 mM in nitrogen residues) also prepared in 150 mM NaCl for 10 min. The cDNA-PEI complexes were transferred to HEK-293T cells and were incubated for 4 h in a serum-starved medium. Then, the medium was replaced by a fresh supplemented culture medium, and cells were maintained at 37 °C in a humid atmosphere of 5% CO_2_. 48 h after transfection, cells were washed, detached, and resuspended in the assay buffer.

### Immunocytochemistry

HEK-293T cells were seeded on glass coverslips in 12-well plates. Twenty-four hours after, cells were transfected with CB_2_-YFP cDNA (1 μg), GluN1-RLuc cDNA (1 μg), and GluN2B cDNA (0.75 μg). Forty-eight hours after, cells were fixed in 4% paraformaldehyde for 15 min and washed twice with PBS containing 20 mM glycine before permeabilization with PBS-glycine containing 0.2% Triton X-100 (5 min incubation). Cells were blocked during 1 h with PBS containing 1% bovine serum albumin. HEK-293T cells were labeled with a mouse anti-RLuc antibody (1/100; Millipore, Darmstadt, Germany) and subsequently treated with Cy3-conjugated anti-mouse (1/200; Jackson ImmunoResearch (red)) antibody (1 h each). The CB_2_R-YFP expression was detected by the YFP’s own fluorescence. Nuclei were stained with Hoechst (1/100 from stock 1 mg/mL; Sigma-Aldrich). Samples were washed several times and mounted with 30% Mowiol (Calbiochem).

Images were obtained in a Zeiss LSM 880 confocal microscope (ZEISS, Germany) with the 40X and 63X oil objectives.

### Bioluminescence resonance energy transfer (BRET) assay

For BRET assay, HEK-293T cells were transiently cotransfected with a constant amount of cDNA encoding for GluN1-RLuc (0.25 μg) and GluN2B (0.15 μg) and with increasing amounts of cDNA corresponding to CB_2_R-YFP (0.25 to 1.25 μg). As a negative control, HEK-293T cells were transiently cotransfected with a constant amount of cDNA encoding for GluN1-RLuc (0.25 μg) and GluN2B (0.15 μg) and with increasing amounts of cDNA corresponding to GHSR1a-YFP (0.25 to 1.5 μg). To control the cell number, sample protein concentration was determined using a Bradford assay kit (Bio-Rad, Munich, Germany) using bovine serum albumin (BSA) dilutions as standards. To quantify fluorescent proteins, cells (20 μg of total protein) were distributed in 96-well microplates (black plates with a transparent bottom) and fluorescence was read in a Fluostar Optima Fluorimeter (BMG Labtech, Offenburg, Germany) equipped with a high-energy xenon flash lamp, using a 10-nm bandwidth excitation filter at 485 nm. For BRET measurements, the equivalent of 20 μg of total protein cell suspension was distributed in 96-well white microplates with a white bottom (Corning 3600, Corning, NY). BRET was determined one minute after adding coelenterazine H (Molecular Probes, Eugene, OR), using a Mithras LB 940 plate reader (Berthold Technologies, DLReady, Germany), which allows the integration of the signals detected in the short-wavelength filter at 485 nm and the long-wavelength filter at 530 nm. To quantify GluN1-RLuc expression, luminescence readings were obtained 10 min after the addition of 5 μM coelenterazine H. MilliBRET units (mBU) are defined as:

$$\mathrm{mBU}=\left[\ \frac{\uplambda_{530}\left(\mathrm{long}-\mathrm{wavelength}\ \mathrm{emission}\right)}{\uplambda_{485}\left(\mathrm{short}-\mathrm{wavelength}\ \mathrm{emission}\right)}-{\mathrm{C}}_{\mathrm{f}}\ \right]\times 1000$$

where C_f_ corresponds to [(long-wavelength emission)/(short-wavelength emission)] for the RLuc construct expressed alone in the same experiment.

### cAMP level determination

The analysis of cAMP levels was performed in HEK-293T cells cotransfected with the cDNA for two subunits of the NMDA receptor, GluN1 (1 μg) and GluN2B (0.75 μg) or/and/or the cDNA for the CB_2_R (1 μg). Similar assays were also performed in primary microglia and primary neurons prepared from wild-type mice or the transgenic APP_Sw/Ind_ AD mice model. In the case of microglia cells were first activated using 1 μM LPS and 200 U/mL IFN-γ (48 h). Two hours before the experiment, the medium was substituted by serum-starved DMEM medium. Cells growing in a medium containing 50 μM zardaverine were distributed in 384-well microplates (2000 HEK-293T cells or 4000 hippocampal neurons or microglial cells per well) followed by the stimulation with the NMDA and/or CB_2_R agonists (NMDA (15 μM) and/or JWH-133 (100 nM)) for 15 min before adding 0.5 μM forskolin or vehicle for an additional 15 min period. When indicated cells were pre-treated (15 min) with the NMDA or CB_2_R antagonists, respectively, MK-801 (1 μM) or SR-144528 (1 μM). Homogeneous time-resolved fluorescence energy transfer (HTRF) measures were performed using the Lance Ultra cAMP kit (PerkinElmer). Fluorescence at 665 nm was analyzed on a PHERAstar Flagship microplate reader equipped with an HTRF optical module (BMG Labtech). A standard curve for cAMP was obtained in each experiment.

### MAP kinase pathway activation is measured by ERK1/2 phosphorylation

Hippocampal neurons, microglial cells, or HEK-293T cells cotransfected with the cDNA for the protomers of the NMDA receptor, GluN1 (1 μg) and GluN2B (0.75 μg), and/or with the cDNA for CB_2_R (1 μg) were plated in transparent Deltalab 96-well microplates. Primary microglial cells were activated by incubating cells with 1 μM LPS and 200 U/mL IFN-γ during 48 h. Two hours before the experiment, the medium was substituted by serum-starved DMEM medium. Cells were treated or not for 10 min with the selective antagonists (MK-801 (1 μM) or SR-144528 (1 μM)) followed by 7 min treatment with the selective agonists (NMDA (15 μM) and/or JWH-133 (100 nM)). Cells were then washed twice with cold PBS before the addition of lysis buffer (15 min treatment). Ten microliters of each supernatant was placed in white ProxiPlate 384-well microplates and ERK1/2 phosphorylation was determined using an AlphaScreen®SureFire® kit (Perkin Elmer) following the instructions of the supplier and using an EnSpire® Multimode Plate Reader (PerkinElmer).

### Detection of cytoplasmic calcium levels

HEK-293T cells were cotransfected with the cDNA for the protomers of the NMDA receptor channel GluN1 (1 μg) and GluN2B (0.75 μg), with thee cDNA for CB_2_R (1 μg) and with the cDNA for the GCaMP6 calcium sensor (1 μg) [47] by the use of PEI method (Section “Cell Transfection”). 48 hours after transfection, HEK-293T cells plated in 6-well black, clear bottom plates, were incubated with Mg^2+^-free Locke’s buffer (154 mM NaCl, 5.6 mM KCl, 3.6 mM NaHCO_3_, 2.3 mM CaCl_2_, 5.6 mM glucose, 5 mM HEPES, 10 μM glycine, pH 7.4). Online recordings were performed right after the addition of agonists. When indicated cells were pre-treated with receptor antagonists for 10 min. Fluorescence emission intensity due to complexes GCaMP6 was recorded at 515 nm upon excitation at 488 nm on the EnSpire® Multimode Plate Reader for 150 s every 5 s at 100 flashes per well.

### Dynamic mass redistribution (DMR) label-free assays

Cell signaling was explored using an EnSpire® Multimode Plate Reader (PerkinElmer) by a label-free technology. Cellular cytoskeleton redistribution induced upon receptor activation was detected by illuminating the underside of the plate with polychromatic light and measured as changes in wavelength of the reflected monochromatic light. The magnitude of this wavelength shift (in picometers) is directly proportional to the amount of DMR. To determine the label-free DMR signal, 10,000 HEK-293T cells cotransfected with cDNAs for the protomers of the NMDA receptor channel, GluN1 (1 μg) and GluN2B (0.75 μg) and/or with the cDNA for the CB_2_R (1 μg). Similar assays were performed using 10,000 primary neurons from wild type or transgenic APP_Sw/Ind_ mice. Transparent 384-well fibronectin-coated microplates were used until obtaining 70-80% confluent monolayers (kept in the incubator for 24 h). Previous to the assay, cells were washed twice with assay buffer (HBSS with 20 mM HEPES, pH 7.15, 0.1% DMSO) and incubated in the reader with assay buffer for 2 h at 24 °C. Hereafter, the sensor plate was scanned and a baseline optical signature was recorded for 10 min before adding 10 μL of selective agonists (NMDA (15 μM) and/or JWH-133 (100 nM)) also dissolved in assay buffer. When indicated cells were pre-treated with antagonists (MK-801 (1 μM) or SR-144528 (1 μM); 10 μL in volume). Real-time DMR responses were monitored for a minimum of 3600 s.

### Proximity Ligation Assay (PLA)

Detection in natural sources of clusters formed by the NMDA and CB_2_ receptors was addressed in slices of in primary hippocampal microglia and hippocampal neurons of wild type mice or the transgenic APP_Sw/Ind_ mice model. When assays were performed in slices they were embedded in O.C.T. compound (OCT; Tissue Tek Products, Ames Division, Miles Laboratories, Inc., Elkhart, IN, USA) to allow cryostat sectioning (Leica CM3050S; 40 μm-thick sections). When using cells, they were grown on glass coverslips, were fixed in 4% paraformaldehyde for 15 min, washed twice with PBS containing 20 mM glycine to quench the aldehyde groups, permeabilized with the same buffer containing 0.05% Triton X-100 between 5 and 15 min and washed with PBS. After 1 h incubation at 37 °C with the blocking solution in a pre-heated humidity chamber, samples were incubated overnight at 4 °C with a mixture of a rabbit monoclonal anti-GluN1 antibody (1/100, ab52177, Abcam, Cambridge, UK) and a mouse monoclonal anti-CB_2_R antibody (1/100, sc-293188, Santa Cruz Biotechnology, TX, USA). Nuclei were stained with Hoechst (1/100 from 1 mg/mL stock; Sigma-Aldrich). The antibodies were validated following the method in the technical brochure of the vendor with fairly similar results. Cells were further processed using the PLA probes detecting primary antibodies (Duolink In Situ PLA probe Anti-Mouse plus and Duolink In Situ PLA probe Anti-Rabbit minus) (1/5 v:v for 1 h at 37 °C). Ligation and amplification were done as indicated by the supplier (Sigma-Aldrich) and cells were mounted using the mounting medium Mowiol (30%) (Calbiochem). To detect red dots corresponding to CB_2_-NMDA-Hets, samples were observed in a Zeiss LSM 880 confocal microscope (ZEISS, Germany) equipped with an apochromatic 63X oil-immersion objective, and 405-nm and 561-nm laser lines. For each field of view, a stack of two channels (one per staining) and 3 *Z*-planes with a step size of 1 μm were acquired. Andy’s algorithm, a specific ImageJ macro for reproducible and high-throughput quantification of the total PLA foci dots and total nuclei, was used for data analysis [48].

### Statistical analysis

The data in graphs are the mean ± SEM (at least *n*=5). GraphPad Prism 9 software (San Diego, CA, USA) was used for data fitting and statistical analysis. One-way ANOVA followed by post hoc Bonferroni’s test were used when comparing multiple values. Experiments performed in samples from transgenic mice and age-matched controls were analyzed independently, i.e., quantitative inter-group differences were not addressed. When a pair of values were compared, Student’s *t* test was used. Significant differences were considered when the *p* value was <0.05.

## Supplementary Information

**Additional file 1: Figure S1.** Signaling in HEK-293T cells expressing NMDA and GHSR1a receptors. HEK-293T cells transfected with the cDNAs for two protomers of the NMDA receptor: GluN1 (1 μg) and GluN2B (0.75 μg) and/or with the cDNA for the GHS-R1a (1 μg), were treated with selective agonists (15 μM NMDA for NMDAR and/or 100 nM Ghrelin for GHS-R1a). When indicated cells were pretreated with selective receptor antagonists (1 μM MK-801 or 1 μM YIL-781 for GHS-R1a). Panels A-C: Intracellular cAMP levels were determined by TR-FRET as described in Methods. As G_i_ coupling was assessed, decreases in cAMP levels were determined in cells previously treated with 0.5 μM forskolin (15 min). Values are the mean ± S.E.M. of 3 independent experiments performed in triplicates. ANOVA Summary: Panel A; F: 90.9, p<0.001, Panel B; F: 52.1, p<0.001 and Panel C; F: 49.6, p<0.001.

## Data Availability

The datasets used and/or analyzed during the current study are available from the corresponding author on reasonable request.
