# Supplementary material for: N-Methyl-D-aspartate (NMDA) and cannabinoid CB2 receptors form functional complexes in cells of the central nervous system: insights into the therapeutic potential of neuronal and microglial NMDA receptors
Source: Alzheimers Res Ther. 2021 Nov 8;13:184. doi: 10.1186/s13195-021-00920-6 (PMC8576920; doi:10.1186/s13195-021-00920-6)
Supplement: Supplementary file 1 — Additional file 1: Figure S1. Signaling in HEK-293T cells expressing NMDA and GHSR1a receptors. HEK-293T cells transfected with the cDNAs for two protomers of the NMDA receptor: GluN1 (1 μg) and GluN2B (0.75 μg) and/or with the cDNA for the GHS-R1a (1 μg), were treated with selective agonists (15 μM NMDA for NMDAR and/or 100 nM Ghrelin for GHS-R1a). When indicated cells were pretreated with selective receptor antagonists (1 μM MK-801 or 1 μM YIL-781 for GHS-R1a). Panels A-C: Intracellular cAMP levels were determined by TR-FRET as described in Methods. As Gi coupling was assessed, decreases in cAMP levels were determined in cells previously treated with 0.5 μM forskolin (15 min). Values are the mean ± S.E.M. of 3 independent experiments performed in triplicates. ANOVA Summary: Panel A; F: 90.9, p<0.001, Panel B; F: 52.1, p<0.001 and Panel C; F: 49.6, p<0.001. [file 13195_2021_920_MOESM1_ESM.pdf]

Supplementary information

N-Methyl-D-Aspartate (NMDA) and cannabinoid CB_2 receptors form functional complexes in cells of the nervous system. Insights into the therapeutic potential of neuronal and microglial NMDA receptors.

Running title. Cannabinoid-NMDA receptor interactions

Rafael Rivas-Santisteban[1,2], Alejandro Lillo[3,4], Jaume Lillo[1,2], Joan-Biel Rebassa[4], Joan S. Contestí[4], Carlos A. Saura[1,3], Rafael Franco[1,2,5*], Gemma Navarro[1,3,4*]

Affiliations:

[1] Centro de Investigación Biomédica en Red sobre Enfermedades Neurodegenerativas, Instituto de Salud Carlos III, Valderrebollo, 5, 28031 Madrid, Spain; carlos.saura@uab.cat

[2] Departament de Bioquímica i Biomedicina Molecular, Universitat de Barcelona, 08028 Barcelona, Spain

[3] Institut de Neurociències, Department de Bioquímica i Biologia Molecular, Universitat Autònoma de Barcelona, Bellaterra, 08193 Barcelona, Spain

[4] Department of Biochemistry and Physiology, Faculty of Pharmacy and Food Science, University of Barcelona, Barcelona, Spain

[5] School of Chemistry. University of Barcelona, Barcelona, Spain.

***Corresponding authors:**

Rafael Franco (rfranco123@gmail.com; rfranco@ub.edu)
School of Biology. Universitat de Barcelona.
Diagonal 643. Prevosti Building.
Barcelona 08028. Spain
Tel +34 934021208

Gemma Navarro (g.navarro@ub.edu)
School of Pharmacy. Universitat de Barcelona
Joan XXIII, 29-31
Barcelona 08028. Spain
Tel +34 934034500

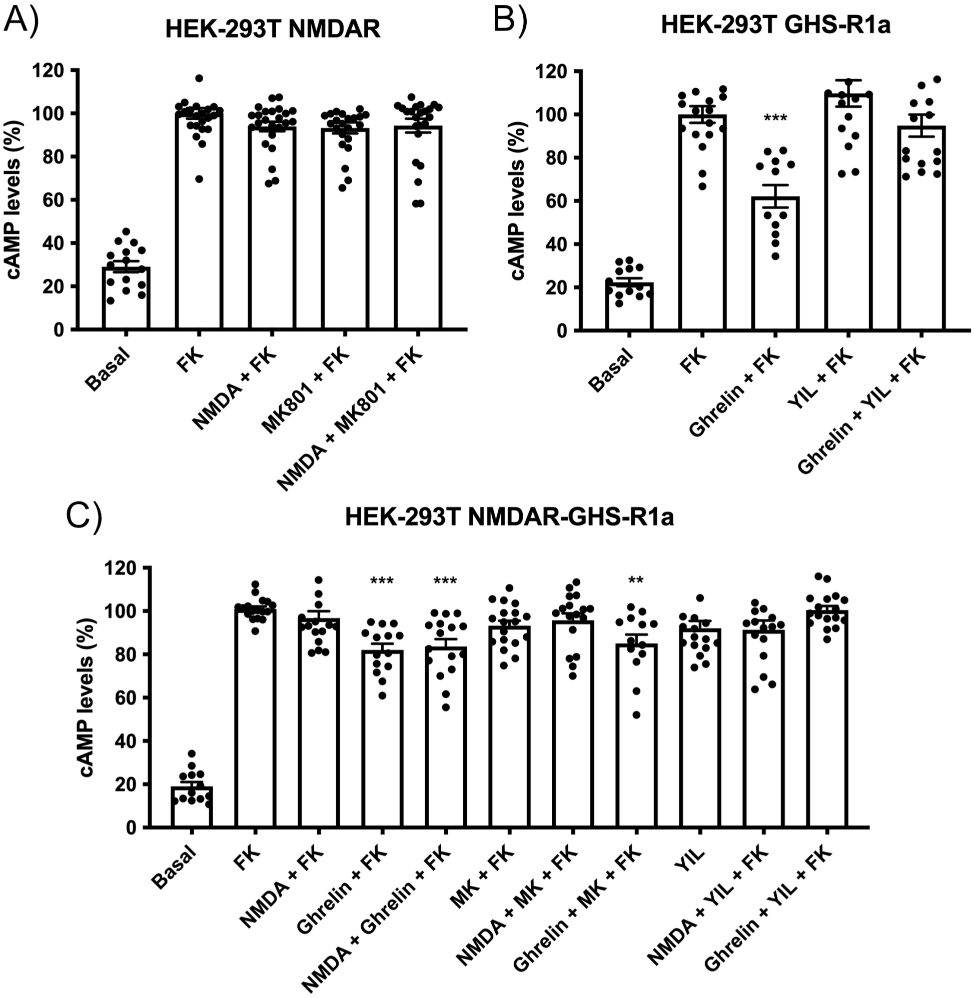

Supplementary figure 1. Signaling in HEK-293T cells expressing NMDA and GHSR1a receptors. HEK-293T cells transfected with the cDNAs for two protomers of the NMDA receptor: GluN1 (1 µg) and GluN2B (0.75 µg) and/or with the cDNA for the GHS-R1a (1 µg), were treated with selective agonists (15 µM NMDA for NMDAR and/or 100 nM Ghrelin for GHS-R1a). When indicated cells were pretreated with selective receptor antagonists (1 µM MK-801 for NMDA or 1 µM YIL-781 for GHS-R1a). Panels A-C: Intracellular cAMP levels were determined by TR-FRET as described in Methods. As G_i coupling was assessed, decreases in cAMP levels were determined in cells previously treated with 0.5 µM forskolin (15 min). Values are the mean ± S.E.M. of 3 independent experiments performed in triplicates. ANOVA Summary: Panel A; F: 90.94, $p<0.001$, Panel B; F: 52.09, $p<0.001$ and Panel C; F: 49,64, $p<0.001$.
